# The Auxin-Response Repressor *IAA30* Is Down-Regulated in Reproductive Tissues of Apomictic *Paspalum notatum*

**DOI:** 10.3390/plants11111472

**Published:** 2022-05-31

**Authors:** Lorena Adelina Siena, Celeste Antonela Azzaro, Maricel Podio, Juliana Stein, Olivier Leblanc, Silvina Claudia Pessino, Juan Pablo Amelio Ortiz

**Affiliations:** 1 Laboratorio de Biología Molecular, Instituto de Investigaciones en Ciencias Agrarias de Rosario (IICAR) CONICET-UNR, Facultad de Ciencias Agrarias, Campo Experimental Villarino, Universidad Nacional de Rosario, Zavalla S2125ZAA, Santa Fe, Argentina; siena@iicar-conicet.gob.ar (L.A.S.); celesteazzaro@hotmail.com (C.A.A.); podio@iicar-conicet.gob.ar (M.P.); jstein@unr.edu.ar (J.S.); pessino@iicar-conicet.gob.ar (S.C.P.); 2 DIADE, Université de Montpellier, IRD, CIRAD, 34394 Montpellier, France; olivier.leblanc@ird.fr

**Keywords:** apomixis, Aux/IAA, auxin, plant reproductive development, *Paspalum notatum*

## Abstract

The capacity for apomixis in *Paspalum notatum* is controlled by a single-dominant genomic region, which shows strong synteny to a portion of rice chromosome 12 long arm. The locus LOC_Os12g40890, encoding the Auxin/Indole-3-Acetic Acid (Aux/IAA) family member *OsIAA30,* is located in this rice genomic segment. The objectives of this work were to identify transcripts coding for Aux/IAA proteins expressed in reproductive tissues of *P. notatum*, detect the *OsIAA30* putative ortholog and analyze its temporal and spatial expression pattern in reproductive organs of sexual and apomictic plants. Thirty-three transcripts coding for AUX/IAA proteins were identified. Predicted protein alignment and phylogenetic analysis detected a highly similar sequence to *OsIAA30* (named as *PnIAA30)* present in both sexual and apomictic samples. The expression assays of *PnIAA30* showed a significant down-regulation in apomictic spikelets compared to sexual ones at the stages of anthesis and post-anthesis, representation levels negatively correlated with apospory expressivity and different localizations in sexual and apomictic ovules. Several *PnIAA30* predicted interactors also appeared differentially regulated in the sexual and apomictic floral transcriptomes. Our results showed that an auxin-response repressor similar to *OsIAA30* is down-regulated in apomictic spikelets of *P. notatum* and suggests a contrasting regulation of auxin signaling during sexual and asexual seed formation.

## 1. Introduction

Seeds in angiosperms can originate following sexual or asexual routes [1]. The sexual path typically involves the double fertilization of the haploid female gametophyte or embryo sac (ES), derived from the single product that survived meiosis (the functional megaspore or FM) of the megaspore mother cell (MMC). During this process, one of the two sperm cells delivered by the pollen fuses with the egg cell to form the diploid zygote that will give rise to the embryo, while the second one fuses with the dihaploid central cell to generate the primary nucleus of the triploid endosperm [1]. This reductional/recombinant/bigametic pathway leads to the generation of genetically and phenotypically variable offspring [2,3]. Alternatively, asexual seed formation (i.e., apomixis) involves the production of seeds carrying embryos that are genetically identical to the mother plant [4]. Apomixis can follow two general mechanisms [4]. In sporophytic apomixis (or adventitious embryony), one to several maternal embryos can arise from somatic cells of the ovule. Successful seed formation requires the fertilization of the sexual ES to generate the endosperm, which is shared by the maternal and sexually produced embryos [5,6]. In gametophytic apomixis, seeds derive from unreduced ESs that originate either from: (a) the MMC after suppressing or modifying meiosis (diplospory) or (b) nucellar cells surrounding the MMC, which develop into apospory initials (AIs) (apospory) [5]. Seed formation follows with the emergence of maternal embryos by parthenogenesis (without fertilization) and the formation of the endosperm autonomously (autonomous apomixis) or after fertilization of the unreduced polar nuclei (pseudogamous apomixis) [7,8]. Typically, the male germline forms viable reduced pollen [9], although meiosis abnormalities have been observed in several apomictic species, including *Paspalum* [10]. Apomixis and sexuality are closely related and not mutually exclusive since both types of reproduction coexist in most apomictic taxa, within the same individual and even in the same ovule [4,8]. During the last two decades, apomictic developments have been considered as deviations of the sexual route, resulting from alterations of the core programs governing megasporogenesis and megagametogenesis [11]. In this view, changes in regulatory pathways that control transcriptional activities could lead to ectopic and/or heterochronic expression of key developmental programs that could mimic apomictic reproduction [12]. A more recent alternative idea stipulates that apomixis and sexuality can be perceived as ancient reproductive phenisms, and the transition from one to the other could possibly be controlled by environmental signals [13]. Gametophytic apomixis is widely spread in the Poaceae, Asteraceae and Rosaceae families but absent from major crop species, except for some subtropical forage grasses and fruit trees [14]. As the trait allows the fixation of heterosis, it is a valuable tool for breeding and crop production. Its manipulation permits full exploitation of hybrid vigor, acceleration of breeding programs and avoidance of the sanitary problems associated with clonal vegetative propagation [7,15,16]. Breeding programs based on apomixis technology are currently being employed in forage grasses of *Brachiaria*, *Panicum*, *Paspalum* and *Poa* genera [17,18,19,20,21].

Apomixis is a genetically controlled trait. In several grass species, a single Apomixis Controlling Locus (ACL) has been identified within non-recombinant heterochromatic regions, presumably after structural genomic rearrangements, including gene migration and/or invasion by transposable elements [7,22]. This complex structure has long hampered the identification of apomixis-controlling genes [23]. In *P. notatum*, the ACL shows a distorted segregation ratio, strong restriction of recombination and heavy cytosine methylation [24]. Comparative mapping revealed that a segment of about 5.8 cm of the rice chromosome 12 long arm is syntenic to the ACL of *P. notatum* and three other *Paspalum* species [24,25,26]. This rice chromosome segment harbors strong candidate genes for apomixis, including LOC_Os12g40890 (Os12g0601300), encoding a member of the AUXIN RESPONSIVE AUX/IAA protein family (OsIAA30) [26]. The homozygous gain-of-function mutation (*iaa16-1*) of the *OsIAA30 Arabidopsis* ortholog (*AtIAA16*, AT3G04730) shows severe growth defects and a decreased anther filament elongation that causes sterility [27].

Auxin plays an essential role in many aspects of plant development [28]. Changes in auxin levels can trigger gene reprogramming through the action of early-response gene families, including Aux/IAAs (AUX/Indole-3-Acetic Acids), ARFs (Auxin Response Factors), SAUR (Small Auxin Up-regulated RNAs) and GH3 (auxin-responsive Gretchen Hagen 3) [29]. The AUX/IAA family negatively regulates auxin-mediated transcription by binding to ARFs through conserved domains (III and IV) occurring in both types of proteins [30]. The degradation of ubiquitin-modified AUX/IAA releases ARFs from inhibition, allowing activation or repression of auxin-responsive genes [31]. During reproductive development, auxins are critical for the somatic-to-germline fate transition, megasporogenesis, female gametophyte development and seed formation [32,33,34]. Particularly, during megagametogenesis, auxin participates in cell fate specification and ES patterning [3,35,36]. Alterations in the expression of auxin-related genes have been associated with apomixis and somatic embryogenesis in several species [23,37]. In *Poa pratensis*, *SOMATIC EMBRYOGENESIS RECEPTOR KINASE (SERK*) signaling may interact with the auxin pathway controlled by *APOSTART* during the induction of aposporous embryo sacs and embryogenesis [38]. In rice, the ectopic expression of *OsBBM1* induces somatic embryogenesis in differentiated tissues through the up-regulation of the core auxin (IAA) biosynthesis genes *YUCCA* [39]. In *P. notatum*, several sRNA and miRNA targeting genes related to auxin metabolism, transport and signaling are differentially regulated during apomictic and sexual development [40]. Moreover, numerous transcripts associated with auxin metabolism, including homologs of Arabidopsis genes involved in IAA synthesis (e.g., *YUCCA 9*, *SUPER ROOT1*-*TYR AMINOTRANSFERASE 2* and *SUPER ROOT2 P450 MONOOXYGENASE*), showed differential regulation between the two reproductive modes [41].

The genetic location of the auxin-response repressor *OsIAA30* in a region syntenic to the *Paspalum* ACL allows raising the hypothesis that auxin-regulatory pathways might play a triggering role in one to several steps of apomictic development in this species. The objective of this work was to explore the possible link between apomixis and auxin signaling in *P. notatum* by conducting a detailed comparative spatio-temporal expression analysis of the putative *OsIAA30* ortholog during sexual and apomictic reproductive development.

## 2. Results

### 2.1. Mining the P. notatum Floral Transcriptome for Homologs of Rice AUX/IAA Members

**Figure 1:**
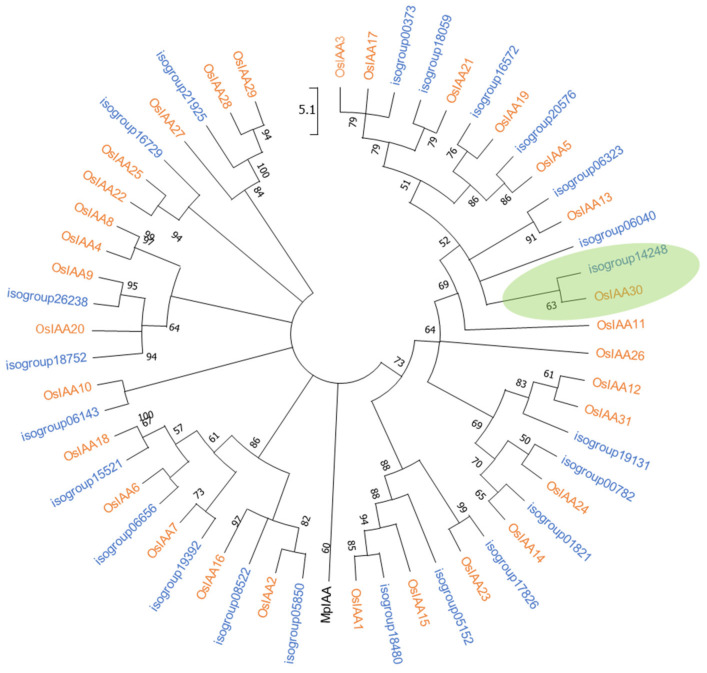
Phylogenetic analysis of AUX/IAA proteins of rice (*O. sativa* var. *japonica*) and of *P. notatum* floral transcriptome. Phylogenic trees were rooted in *Marchantia polymorpha* Aux/IAA (MpIAA; accession AKJ88117). A neighbor-joining circular tree was built by using Mega X software [46]. Branch lengths show evolutionary distances based on the number of amino acid differences per sequence. Bootstrap support values (1000 replicates) are indicated along branches. The shadowed bubble indicates the localization of OIAA30 and its putative *Paspalum* ortholog.

**Figure 2:**
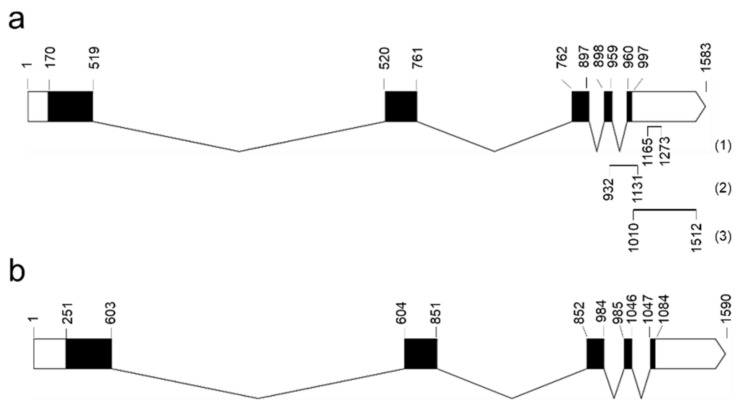
Predicted structure of *PnIAA30* gene (**a**) compared to *OsIAA30* (**b**). cDNA sequences were aligned to the corresponding genomic sequences of *Sorghum bicolor* (Sb08g020580) and *Oryza sativa* (Os12g0601300), respectively. Black and white boxes indicate exons and UTR regions, respectively. Lines indicate introns. Segments at the 3′ end of *PnIAA30* indicate the fragments amplified in qRT-PCR experiments (1, 2) and the fragment used as a probe in in situ hybridization assays (3).

We queried a previously available *P. notatum* floral transcriptome [42] using the sequences of 31 AUX/IAA rice proteins (OsIAA1-31) [43] and identified 33 unique transcripts (isotigs) that assembled into 23 isogroups (1 to 4 isotigs per group) (Table S1, pages 1 and 2). The alignments of the predicted amino acid sequences (ranging 155–387 aa and 16.44–41.05 kDa) revealed that all of them contained the four conserved Aux/IAA domains (PF02309, CL0072), including: domain I (D/ELXLXL), responsible for target genes repression and recruitment of the TOPLESS (TPL) corepressor; domain II (GWPPV) or “degron”, conferring instability and causing rapid degradation of Aux/IAA when interacting with F-box TIR1/AFB proteins; and C-terminal domains III and IV, involved in homo and/or heterodimerization with Aux/IAA proteins and/or with ARFs, respectively [43] (Figure S1a,b). Then, we inferred phylogenetic relationships among rice and *P. notatum* Aux/IAA proteins from a full-length alignment of the 31 rice members described previously [44] and one representative of each *P. notatum* (iso)group. A total of 11 clusters were identified (Figure 1). Phylogenetic analyses defined at least 22 putative *P. notatum* orthologs supported by bootstrap values of over 50%. Interestingly, in 8 out of the 10 highly supported rice sister-pair proteins described by Jain et al. (2006) [44], 2 or more *P. notatum* members were also grouped, indicating possible gene duplications. Four rice proteins (OsIAA4, OsIAA8, OsIAA11 and OsIAA26) did not cluster with any of the *P. notatum* sequences, suggesting that putative orthologs have minor or no expression in floral tissues or are directly absent from the genome (Figure 1). OsIAA30 (277 amino acids), encoded by LOC_Os12g40890, grouped with isogroup14248 (composed of a single transcript, i.e., isotig33023), with a similarity value of 77.24% (E-value = 730 × 10^−128^) (Table S1, page 1). Isogroup14248 was thus considered to represent the product of the *OsIAA30* ortholog gene and, accordingly, named *PnIAA30*. Transcripts expressed in sexual (NCBI GFNR01018320.1) and apomictic (NCBI GFMI02021163.1) flowers were highly similar (ID > 99.60%) with few differences, i.e., one 3-bp INDEL at the 5′ region and a few scattered SNPs (Figure S2a). Both transcripts encoded nearly identical proteins (%ID = 99.64) of 276 and 275 amino acids, respectively (Figure S2b), that showed 74.5% shared identity with OsIAA30. Accordingly, both sequences were assumed to be products of the alleles of the *PnIAA30* gene. Finally, the exon/intron structure was investigated based on the genomic sequence of the ortholog of *Sorghum bicolor* (Sb08g020580), a species phylogenetically associated with *P. notatum* [45] and, as shown in Figure 2a, exon/intron boundaries similar to that of the rice gene (Figure 2b) were predicted for the consensus *PnIAA30* transcript.

### 2.2. Expression Analyses of PnIAA30 in Reproductive Tissues

**Figure 3:**
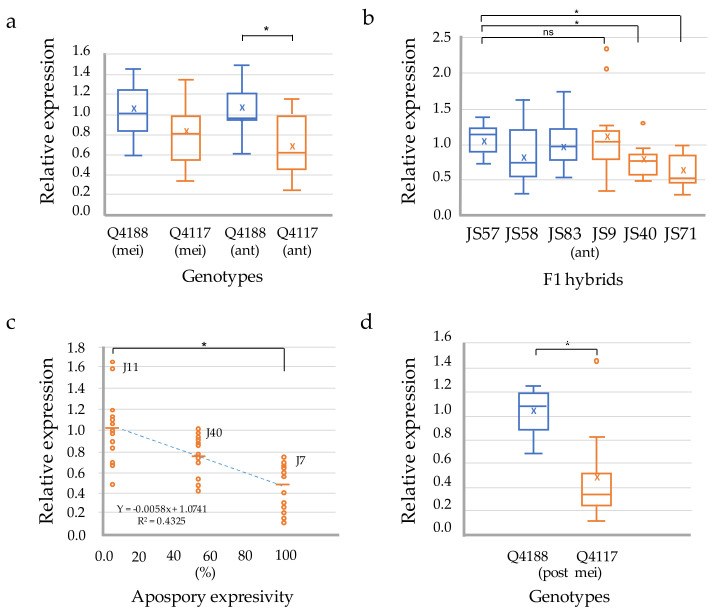
Expression analyses of *PnIAA30* transcripts in reproductive organs of sexual and apomictic *P. notatum* plants. (**a**) qRT-PCR determination in spikelets of sexual (Q4188) and apomictic (Q4117) genotypes collected at meiosis (mei) and anthesis (ant). (**b**) Relative expression in three sexual and three apomictic full-sib F_1_ hybrids derived from a Q4188 vs. Q4117 cross. (**c**) Linear regression between *PnIAA30* expression levels (*Y*-axis) and the expressivity of apospory in three genotypes (*X*-axis). (**d**) qRT-PCR determination in spikelets at two–three days after anthesis. In (**a**,**b**,**d**), boxplots show the third quartile (75th percentile) and the first quartile (25th percentile) range of the data, the median (line in box) and the mean (x). Upper and lower whisker caps indicate the 95th and 5th percentiles, and outliers are indicated with circles. * denotes significant differences at *p*-value ≤ 0.05 compared to the sample used as the calibrator.

The relative expression levels of PnIAA30 transcripts during the reproductive development of sexual (Q4188) and apomictic (Q4117) genotypes were analyzed by qRT-PCR using specific primers designed to amplify a segment in the 3′ region absent from the other members of the AUX/IAA family (Figure 2a). qRT-PCR assays revealed a similar relative expression at the stage of meiosis in sexual and apomictic samples (*t*-test *p* = 0.073), while a significantly higher expression was detected in the sexual sample in comparison to the apomictic one at anthesis (*t*-test *p*= 0.010) (Figure 3a; Table S2). Next, we assessed the expression of *PnIAA30* at anthesis in florets of three sexual (JS57, JS58 and JS83) and three apomictic (JS9, JS40 and JS71) F_1_ hybrids derived from a cross between Q4188 and Q4117 genotypes [47]. Two of the apomictic hybrids (JS40 and JS71) showed significantly lower values than the sexual sample (JS57) used as the calibrator (Figure 3b) (Table S2), but the mean comparison of the sexual and apomictic samples showed no difference (*p* = 0.171) (Table S2). In addition, we measured *PnIAA30* expression levels in genotypes exhibiting different apospory expressivity (i.e., proportion of ovules bearing one or more aposporous ESs). In this case, we used cDNA samples prepared from inflorescences of three full-sib hybrids showing 100% (J7), 56% (J40) and 3% (J11) of aposporous ovules at anthesis (Carlos Acuña, IBONE, Corrientes, Argentina; personal communication). This analysis revealed a significant negative correlation (R^2^ = 0.4325; *p* < 0.001) between *PnIAA30* expression and apospory expressivity (Figure 3c) (Table S2). *PnIAA30* expression at early embryogenesis (two–three days after anthesis) also revealed a significant difference (*p* = 0.001) between sexual and apomictic samples (Figure 3d; Table S2). On the other hand, no difference in *PnIAA30* expression was detected between Q4188 and Q4117 in sporophytic tissues, such as leaves (*p* = 0.498) and roots (*p* = 0.191) (Figure S3, Table S2).

### 2.3. In Situ Localization of PnIAA30 Expression

To gain further insight into *PnIAA30* expression, we assessed the spatial profile by performing in situ hybridization experiments on florets containing ovules at the stages of premeiosis/meiosis and anthesis. A specific 503-bp fragment located at the 3′ end of the transcript, at positions 1010–1512 within the consensus sequence, was used as a probe (Figure 2a). The fragment amplified from the apomictic genotype showed >99.6% identity (E-value = 0.00) with NCBI accessions GFNR01018320.1 (*PnIAA30*_sex) and GFMI02021163.1 (*PnIAA30*_apo) and no homology with other transcripts expressed in the *P. notatum* floral transcriptome, thus confirming its high specificity. 

**Figure 4:**
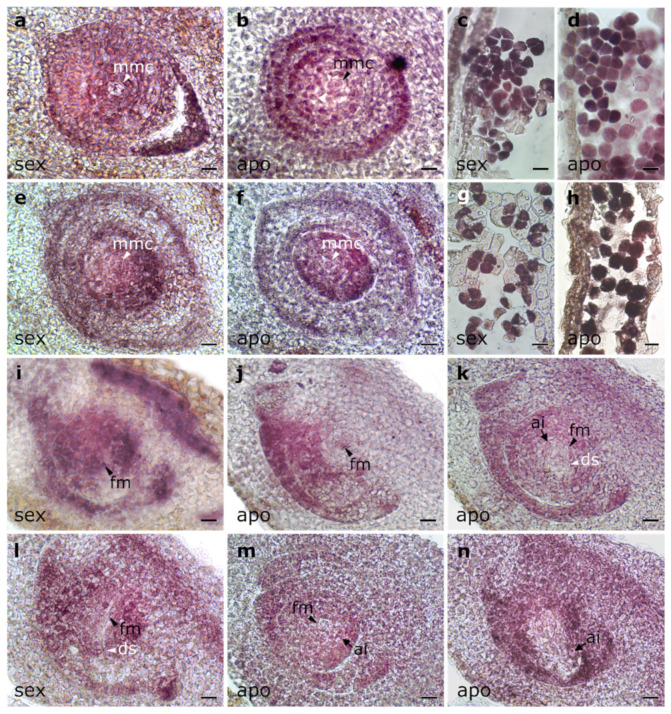
RNA in situ hybridization of *PnIAA30* transcripts in reproductive organs of sexual (Q4188) and apomictic (Q4117) *P. notatum* individuals. (**a**–**d**) Hybridization with the antisense probe in cross-sections of ovules at the stage of MMC and anthers bearing tetrads of microspores of sexual (**a**,**c**) and apomictic (**b**,**d**) plants. (**e**–**h**) Hybridization with the sense probe in cross-sections of ovules at the MMC stage and anthers with tetrads of microspores in sexual (**e**,**g**) and apomictic (**f**,**h**) individuals. (**i**–**k**) Sagittal sections of ovules of the sexual (**j**) and apomictic (**j**,**k**) plants at the stage of FM, hybridized with the antisense probe. (**j**,**k**) Adjacent sections of the same ovule from Q4117. (**l**–**n**) Sagittal sections of ovules of sexual (**l**) and apomictic (**m**,**n**) genotypes hybridized with the sense probe. ai: apospory initials, apo: ovules of Q4117, ds: degenerating spores, fm: functional megaspore cell. mmc: megaspore mother cell, sex: ovules of Q4188. Bars: 10 µm.

Hybridization performed with the antisense probe (detecting the coding strand) in ovules having an MMC (i.e., before female meiosis) had signals restricted to nucellus and integuments in both sexual and apomictic genotypes (Figure 4a,b) that contrasted with the surrounding tissues (Figure S4). In sexual ovules, the MMC lacked signal (Figure 4a), while in apomictic ovules, it showed staining similar to the contiguous nucellar cells (Figure 4b). In anthers, microspores of both genotypes showed strong signals (Figure 4c,d and Figure S4). Hybridizations with a sense probe (which detects transcript antisense strand) showed antisense expression in MMCs, nucellar cells, integuments (Figure 4e,f and Figure S5) and microspores (Figure 4g,h) of both genotypes.

Analyses using antisense probes at the stage of FM also showed hybridization signals in nucellar cells and in integuments of both sexual and apomictic genotypes (Figure 4j,k). However, while the FM showed hybridization levels similar to the surrounding somatic cells in sexual ovules (Figure 4i), the FM in apomictic ovules appeared almost without hybridization (Figure 4j,k). Chalazal regions also showed contrasted hybridization patterns with higher signals in the sexual genotype (compare Figure 4j–l). The sense probe revealed the presence of antisense transcripts in nucellar cells and integuments of both Q4188 and Q4117 (Figure 4l,m). However, in contrast to the FM in ovules of Q4188 that showed hybridization (Figure 4l), both the FM and apospory initials (AI) lacked hybridization signals in ovules of the apomictic genotype (Figure 4m,n). 

**Figure 5:**
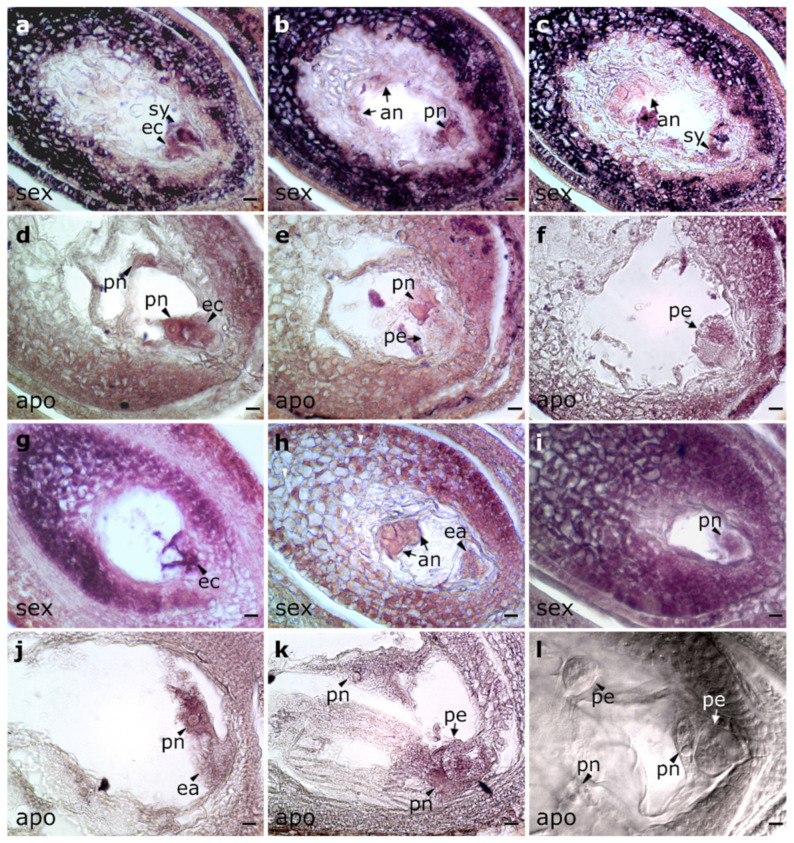
RNA in situ hybridization of *PnIAA30* transcripts in ovaries from sexual (Q4188) and apomictic (Q4117) *P. notatum* at anthesis. (**a**–**f**) Hybridization with the *PnIAA30* antisense probe of sexual (**a**–**c**) and apomictic (**d**–**f**) genotypes. Notably, in the apomictic ovule, a proembryo showed almost no *PnIAA30* hybridization signal ((**e**,**f**), adjacent sections of the same ovule). (**g**–**k**) Hybridization with *PnIAA30* sense probe in sexual (**g**–**i**) and apomictic (**j**,**k**) ovaries. (**l**) DIC reference image for apomictic ovules at anthesis bearing multiple embryo sacs, parthenogenetic embryos and unfertilized polar nuclei. an: antipodal cells, ea: egg apparatus ec: egg cell, pn: polar nuclei, pe: pro-embryo, sy: synergid cells. Bars: 10 µm.

At anthesis, the antisense probe revealed a more contrasting pattern between sexual and apomictic genotypes. In Q4188, ovaries displayed strong signals in integuments, nucellus, the egg apparatus and polar nuclei (Figure 5a–c). In Q4117, integuments and the egg apparatus stained faintly (Figure 5d–f). Interestingly, in apomictic ovules, parthenogenetic proembryos showed a very low signal (Figure 5f). Hybridizations with the sense probe also showed differences between genotypes. While strong hybridization was detected in the nucellus, integuments and reproductive cells of the sexual ovules (Figure 5g,i), only a pale hybridization was observed in polar nuclei and parthenogenetic embryo of apomictic ovules (Figure 5j,k). Finally, note that in all assays, negative controls showed no NBT/BCIP staining (Figure S6).

Overall, these results indicate expression pattern differences for *PnIAA30* (sense and antisense transcripts) in ovules of sexual and apomictic genotypes at both stages tested (premeiosis/meiosis and anthesis). The results are in agreement with qRT-RCR analyses, which detected higher expression levels at anthesis in sexual ovules compared to apomictic ones. Interestingly, proembryos in apomictic genotypes showed low expression levels for both sense and antisense transcripts.

### 2.4. sRNA Might Be Regulating PnIAA30 Expression

**Figure 6:**
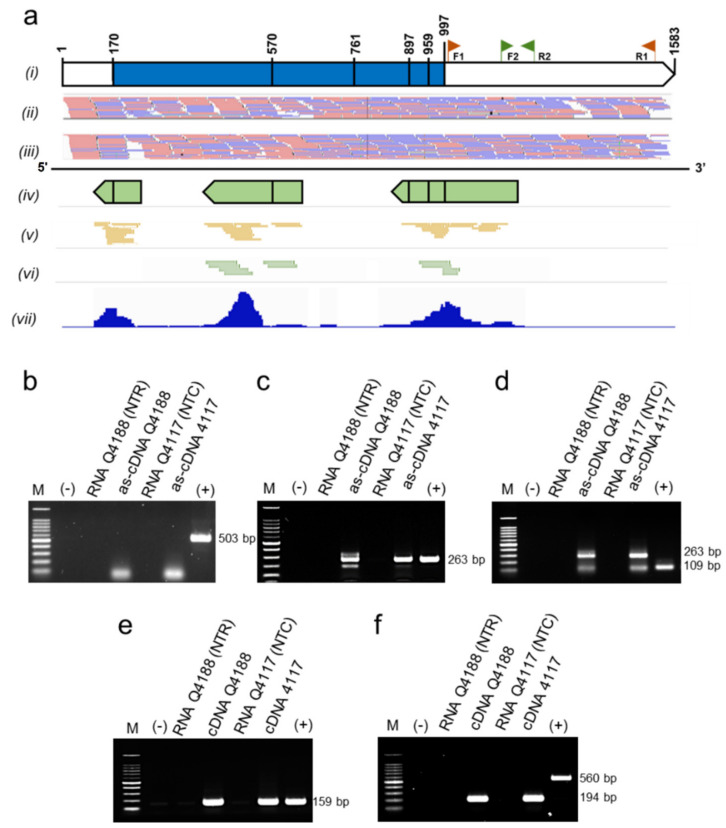
Validation of *PnIAA30* antisense strand expression. (**a**) Sense and antisense RNA reads from sexual (Q4188) and apomictic (Q4117) samples mapped to *PnIAA3*0 transcript: (***i***) diagram of the sense transcript showing the locations of intron-exon junctions and primers pairs used for PCR amplification: F1 (IAA30_probeF), R1 (IAA30_probeR), F2 (RTPnIAA30F) and R2 (RTPnIAA30R), (***ii***,***iii***) coverage of the sense reads from sexual (***ii***) and apomictic (***iii***) samples, (***iv***) diagram of the consensus antisense reads mapping to the transcript, (***v***,***vi***) coverage of antisense reads in the sexual and apomictic samples, respectively, (***vii***) histograms of the antisense reads from both sexual and apomictic samples mapping to *PnIAA30*. (**b**–**d**) Agarose gels showing PCR amplifications from the *PnIAA30* antisense strand-specific cDNA (as-cDNA) generated using primer F1. (**b**) Amplification with primers F1 and R1. (**c**) Amplification with primers F1 and R2. (**d**) Amplification with primers F2 and R2. (**e**,**f**) Agarose gels showing control PCR amplifications for *β*-*tubulin* (β-tubulinF and β-tubulinR) and *GPDH* (GPDHF and GPDHR), respectively. All primers used are listed in Table S4. M: molecular weight marker; (−) negative (water) control, NTR: non transcribed RNA, (+) positive control (genomic DNA from Q4117).

Since antisense transcripts were detected during in situ hybridization experiments, we hypothesized that a regulatory mechanism involving RNA molecules might be operative to control *PnIAA30* expression. To check this possibility, we first queried the list reported for transcripts differentially targeted by sRNA during sexual and apomictic development in *P. notatum* [40] and identified a non-coding transcript (isotig26528) similar (score 68, E-value = 4 × 10^−15^, ID% 91) to *OsIAA30* [40]. Alignment of isotig26528 and *OsIAA30* showed a region of 47 nt with a perfect match, which, in the miRBase (http://www.mirbase.org/, accessed on 15 December 2021), showed homology (Score 99, E-value = 0.024) to the *Brachypodium distachyon* miR7768b stem-loop [48]. Moreover, a search in the recently delivered *P. notatum* sense/antisense Illumina TruSeq/HiSeq floral transcriptomes [41] detected several transcripts (i.e., TRpn_30859, TRpn_56938, TRpn_91884, TRpn_120368, TRpn_179711 and TRpn_197389) with homology to AUX/IAA sequences targeted by antisense reads (see Podio et al. 2021, Supplementary Table S4) [41]. Based on these data, we mapped the sense and antisense reads expressed in the sexual and apomictic floral transcriptomes (GenBank GIUR00000000.1) over *PnIAA30*. The assay revealed homogeneous coverage along the transcript with the sense reads derived from both sexual and apomictic genotypes (Figure 6a(*ii*,*iii*)) and partial coverage of the antisense reads in both samples, overlapping exons and splice junctions (Figure 6a(*iv*–*vi*)). Interestingly, the 5′ region of the transcript appeared targeted by antisense reads derived only from the sexual genotype (Figure 6a(*v*)). 

In order to confirm the presence of the antisense reads in vivo, we then carried out PCR assays using an antisense strand (as)-specific cDNA from sexual and apomictic samples as a template. To generate this as-cDNA, we used a primer complementary to the antisense strand of *PnIAA30* (F1), located at the 3′ end of the consensus transcript (Figure 6a(*i*)). This as-cDNA was used as a template in PCR reactions to amplify the following: (1) a fragment of 503 pb coincident with the sequence used as a probe in the in situ hybridization experiments (amplified with primers F1-R1); (2) a segment of 263 bp, amplified with primers F1-R2; and (3) a segment of 109 bp coincident with the sequence amplified in qRT-PCR analyses (amplified with primers F2-R2) (Figure 6 a(*i*),b–f). The 503-bp fragment failed to amplify in all samples (Figure 6b), an outcome in agreement with the mapping results, which showed the absence of antisense reads at the 3′ end of *PnIAA30* transcript where primer R1 anneals (Figure 6b). On the contrary, the 263-bp fragment was amplified from both the sexual and the apomictic samples (Figure 6c). Moreover, the 109-bp fragment coamplified together with the 263 pb expected for the primer combination F1-R2, because copies of the F1 primer were present in the amplification mix as F1 was part of the as-cDNA synthesis reaction (Figure 6d). During the as-cDNA synthesis, sense-specific primers matching the *β-tubulin* and *GDPH* genes were added to generate internal control cDNAs (see Section 4). The amplification of these *β-tubulin* and *GDPH* controls from the sense-specific cDNA template showed the expected products (Figure 6e,f). These results confirmed the expression of antisense transcripts associated with *PnIAA30* in reproductive tissues of *P. notatum* and indicated that the antisense transcripts corresponded to particular regions of the sequence. 

### 2.5. In Silico Analysis of Putative PnIAA30 Interactors

**Figure 7:**
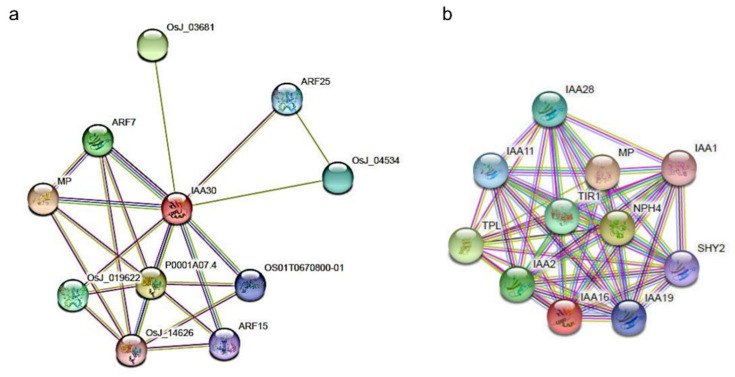
Networks of protein–protein interactions of OsIAA30 (**a**) and AtIAA16 (**b**) as predicted by the STRING database (https://string-db.org/, accessed on 21 January 2022). The name and annotation of each interactor are indicated in Table S3. Each node represents all the proteins produced by a single, protein-coding gene locus. Types of interaction between proteins are indicated by colors (see STRING database references).

**Figure 8:**
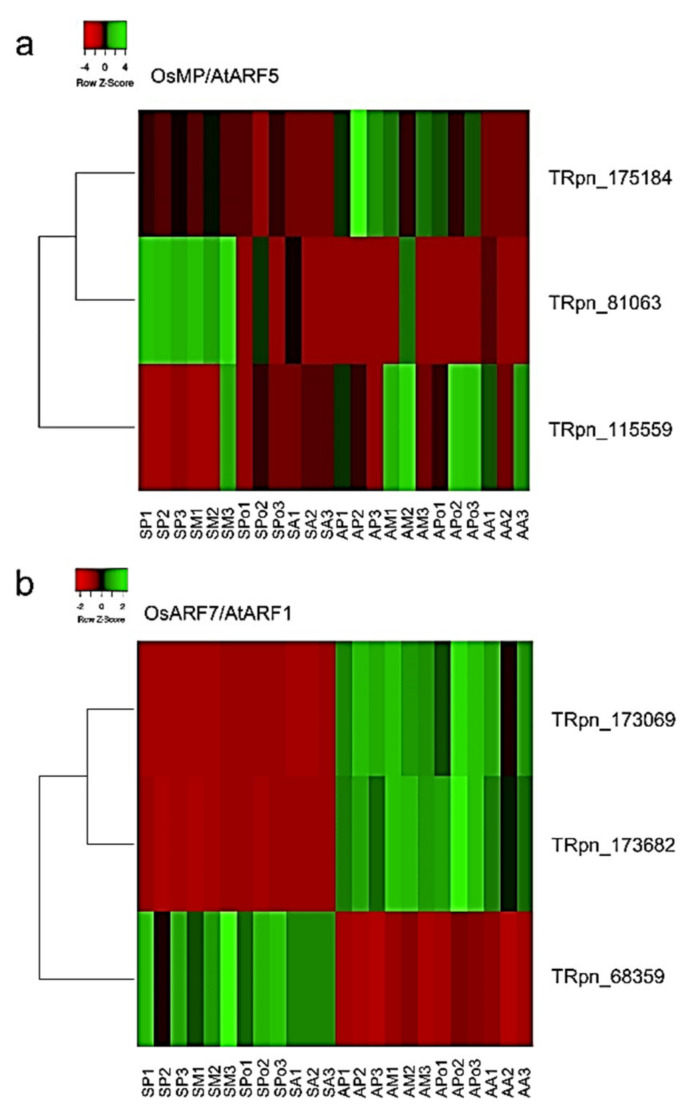
Heatmaps for expression of sense transcripts homologous to OsMP/AtARF5 (**a**) and OsARF7/AthARF1 (**b**) during sexual and apomictic developments in *P. notatum*. The graphs were generated using the normalized numbers of reads detected for each transcript at each developmental stage, as determined by Podio et al. 2021, BMC Genom. 2021, 22, 185 [41]. SP1−3; SM1−3; SPo1−3 and SA1−3: sexual libraries (in triplicate) at the stages of premeosis, meiosis, postmeiosis and anthesis, respectively. AP1−3; AM1−3, Apo1−3, AA1−3: apomictic libraries (in triplicate) at the stages of premeosis, meiosis, postmeiosis and anthesis, respectively. The name of the transcript included in the analysis is shown on the right. Color expression range is indicated at the top left.

In order to detect PnIAA30 interactors expressed during sexuality and apomixis, we carried out an in silico analysis by combining the use of the STRING protein–protein interactions database [49] and the *P. notatum* quantitative floral transcriptome [41]. First, we used STRING to search for interactors of IAA30 and IAA16 in rice (*Oryza sativa* L.) and *Arabidopsis*, respectively. Then, we identified their homologs in the *P. notatum* floral transcriptome and searched for their expression patterns. The initial search in the rice database showed significant interactions (scores values > 0.600) between OsIAA30 and orthologs to *Arabidopsis* ARFs (ARF1, ARF3, ARF5, ARF6, ARF7), TRANSPORT INHIBITOR RESPONSE 1-like (TIR1), Protein AUXIN SIGNALING F-BOX 2 (AFB2) and the PEPTIDYL-PROLYL CIS-TRANS ISOMERASE FK506 (Figure 7; Table S3, page 1). The same analysis carried out using AtIAA16 also detected interactions with ARF5, ARF7 and TIR1 (score > 0.700) and revealed additional targets, including six AUX/IAA proteins (IAA1, IAA2, IAA3, IAA11, IAA19 and IAA28) and TOPLESS, a member of the transducing WD-40 repeat family protein [50] (Figure 7; Table S3, page 1). As expected, most interactors were related to IAA metabolism and early auxin responses. Next, we recovered from the *P. notatum* quantitative floral transcriptomes the homologs of the OsIAA30 (TBLASTn searches) and AtIAA16 (annotations of Podio et al., 2021 [41]) interactors (Table S3, page 2). This search revealed that all predicted interactors, excepting IAA1, 2, 11, 19 and 28, have representatives in the floral transcriptome of *P. notatum*. A subgroup of them, including ARFs 1, 3, 5, 6 and 7, Aux/IAA3, TIR1, TOPLESS, FK506 and AFB2, showed differential expression between sexual and apomictic transcriptomes for at least one developmental stage [41] (Table S3, page 3). Heatmaps of the transcriptomic profile across reproductive developmental stages for the PnARF1 and PnARF5 illustrate the difference between the auxin-dependent transcriptional landscape in sexual and apomictic plants (Figure 8). These results show that several genes related to the IAA30 function are differentially regulated during sexual and apomictic development in *P. notatum*.

## 3. Discussion

Apomixis in *P. notatum* is controlled by a single dominant locus (ACL) that provides capacity for apospory, parthenogenesis and pseudogamy [24]. The presence of coding and non-coding (pseudogenes) sequences, repetitive elements and cytosine methylation are common features of *Paspalum* spp. ACLs [45,51,52]. This genomic region can be considered a gene cluster, which could have evolved after gene duplication followed by neo or sub-functionalization and sequence rearrangement [45]. Gene expression within such clusters is thought to be coordinated at the chromatin level and associated with repressive marks [53]. Since only the ACL is responsible for apomictic reproduction, it must harbor genic or non-genic factors causing the phenotype. Unfortunately, the absence of recombination around the ACL impedes the use of mapping strategies to identify candidate sequences. Therefore, the selection of candidates through comparative genomics with model species is a valuable strategy for deciphering the molecular mechanism governing apomixis [10]. Under this rationale, we focused our work on the analysis of the *P. notatum* homolog of a member of the auxin-response Aux/IAA repressive family, OsIAA30 (LOC_Os12g40890), which is located within a rice chromosome segment syntenic to the *Paspalum* spp. ACLs. 

Aux/IAA repressors play a crucial role in auxin signaling by interacting with ARFs and preventing the targeting of auxin-responsive genes at low hormone concentrations [54]. High auxin levels induce Aux/IAA ubiquitination through interactions with the activated (hormone-coupled) TRANSPORT INHIBITOR RESPONSE 1/AUXIN SIGNALING F-BOX (TIR1/AFB) nuclear receptors and degradation via the 26S proteasome. Then, the released ARFs regulate the expression of auxin-responsive genes [54]. Large AUX/IAA gene families were reported in plants, e.g., 29 members in *A. thaliana* and 31 in rice (see review of Luo et al. 2018 [29]). In our survey, we identified 23 members, most of which (14) are represented by a single transcript (isotig). All sequences encode full proteins containing the four typical AUX/IAA conserved domains; thus they may be functional. Clustering analysis detected 22 putative rice orthologs, 16 of them grouping with 8 out of 10 Aux/IAA sister-pairs (derived from gene duplications) of rice [44]. None of the *Paspalum*-predicted proteins grouped with OsIAA4, OsIAA8, OsIAA11 or OsIAA26, possibly due to a poor representation or lack of transcripts in the reference floral transcriptome, an observation coincident with previous reports [44]. Phylogenetic analysis established that OsIAA30 grouped with the protein encoded by isogroup14248, which comprises a single transcript (isotig32033) and presents nearly identical forms in sexual and apomictic transcriptomes. Based on clustering and high sequence similarity with OsIAA30/AtIAA16, we conclude that isogroup14248 corresponds to the *OsIAA30* ortholog and thus was named *PnIAA30*.

Quantitative expression analyses in reproductive tissues revealed reduced levels of *PnIAA30* transcripts in spikelets of apomicts compared to those of sexual individuals at the stage of anthesis. This trend was confirmed in F_1_ hybrids showing a negative correlation between *PnIAA30* expression and apospory expressivity. Moreover, down-regulation was detected at two–three days post-anthesis, when parthenogenesis is observed in some aposporous embryo sacs. In certain apomicts, the simple application of synthetic auxin a couple of days before pollination (the “auxin test”) induces parthenogenesis in embryos, despite the failure of endosperm development [55]. This procedure was used for estimating the potential for apomixis in *Poa pratensis* [55,56,57]. Moreover, in maize, zygotic genome activation coincides with both a significant up-regulation of genes involved in auxin biosynthesis and signaling and a general decrease in Aux/IAA gene expression [58]. Moreover, it is well known that the core regulatory components of auxin biosynthesis and signaling are involved in plant somatic embryogenesis (reviewed by Wójcik et al. 2020) [37]. Recently, Khanday et al. (2020) [39] proposed that *OsBBM1* (a member of AP2 transcription factor’s family) promotes somatic embryogenesis in rice by increasing auxin metabolism through the up-regulation of *OsYUCCA* genes. The paternal expression of *OsBBM1* in unreduced egg cells of the MiMe genotype was used to induce parthenogenesis of embryos in synthetic apomixis in rice [59]. Based on this large body of data, it is possible that an ACL-directed down-regulation of auxin-response repressors in apomictic ovules might be favoring the parthenogenetic development of maternal embryos in *P. notatum*.

The specific location of *PnIAA30* expression in ovules was determined by in situ hybridization experiments. Before female meiosis, sexual ovules expressed coding transcripts in somatic cells (nucellus and integuments) but not in the cells specified for the female germline (MMCs), while in apomictic ovules, hybridization signals were observed in both the somatic cells and MMCs. Meanwhile, we detected antisense expression at similar levels in both sexual and apomictic ovules. After meiosis, the FM and the surrounding somatic cells of apomictic ovules showed a low expression of the sense and antisense transcripts in clear contrast with sexual ovules. Recently, an attractive model by which non-coding antisense transcripts control the switch from mitosis to the meiosis of the archesporial cell by inhibiting the expression of *OsIME4* sense transcripts in rice has been proposed [60]. A similar mechanism could be acting in apomictic ovules provoking changes in the expression pattern of *PnIAA30* coding transcripts that could mediate both FM and nucellar cells reproductive fate. It is worth noting that *PnIAA30* showed considerable expression levels in microspores of sexual and apomictic genotypes, indicating that it might be essential to male reproductive development but expressed equally in both reproductive types. At anthesis, an even more contrasting expression pattern between apomictic and sexual plants was detected. Integumental cells, nucellar cells, the egg apparatus and polar nuclei of sexual ovules showed strong expression of the *PnIAA30* coding strand, whereas faint expression was detected in apomictic ovules. A similar expression localization was observed for the *PnTGS1*-like gene, a putative apospory repressor [61]. At this stage, the antisense strand was evident in sexual ovules. The expression pattern suggests that, before pollination, aposporous ES are free from the auxin-repressor *PnIAA30* and thus capable of responding to auxin signaling. In addition, the reduction of *PnIAA30* expression detected in aposporous ovules agrees with the behavior of several coding and non-coding sequences located within the ACL of *P. simplex*, which shows down-regulation in ovules [45,62]. Moreover, in a recent work, the antisense down-regulation of the *TGS1*-like gene, which is expressed in nucellar tissues of sexual individuals, induced the formation of an aposporous-like embryo in the species [63].

Finally, to disclose possible molecular pathways associated with *PnIAA30* down-regulation during the apomictic development, an integrative analysis of differentially expressed interactors was carried out by using the STRING database [49] and the quantitative sense and antisense floral transcriptome reported by Podio et al. (2021) [41]. As the biological material used for the transcriptome construction originated from two highly heterozygous tetraploid individuals, the presence of several allelic variants derived from each genotype, also paralogs and/or splicing variants were expected. Out of the ten predicted IAA30/IAA16 interactors, all but one showed one or more variants differentially expressed in sexual and apomictic ovules, suggesting differences in auxin signaling.

In summary, our work provides evidence for the down-regulation of an auxin-response repressor associated with the ACL in spikelets of apomictic *Paspalum* genotypes. Thus, although other genes located in this region could be equally involved in apomictic development, *PnIAA30* arises as a good candidate for further functional analysis aimed at deciphering the mechanistic relationships between auxin signaling and asexual seed formation.

## 4. Materials and Methods

### 4.1. Plant Material

The tetraploid (2n = 4x = 40) *Paspalum notatum* plants used in this study include: Q4117, a natural apomictic accession [64]; Q4188, an experimentally obtained fully sexual tetraploid genotype [65]; six F_1_ hybrids from a mapping population derived from a cross between Q4188 and Q4117 that reproduced either sexually (#57, #58, #83) or apomictically (#9, #40 and #71) [47]. Moreover, three full-sib hybrids derived from a cross between Q4205, a fully sexual female progenitor [65], and a facultative apomictic male progenitor, Q4064 [66], showing different apospory expressivities J11 (3%), J40 (40%) and J7 (100%) (Carlos Acuña; personal communication) were included. All materials belong to the living *Paspalum* germplasm collection of the Instituto de Botánica del Nordeste, (IBONE-CONICET-UNNE), Corrientes, Argentina. Vegetative replicates of each plant were grown in pots at the Instituto de Investigaciones en Ciencias Agrarias de Rosario (IICAR, CONICET-UNR), Zavalla, Argentina, and maintained in neighboring plots to guarantee identical growing environmental conditions.

### 4.2. Identification and Analysis of Transcripts Homologous to Rice AUX/IAA Genes

*P. notatum* transcripts with homology to rice Aux/IAA genes expressed during the sexual and apomictic developments were retrieved from the reference floral transcriptome of the species available at the NCBI BioProject PRJNA330955 [42]. Amino-acid sequences of the 31 rice Aux/IAA proteins (OsIAA1-31) [44] were obtained for the Rice Genome Annotation Project (http://rice.plantbiology.msu.edu/, accessed on 16 September 2020) [67] and used as queries in TBLASTN searches (https://blast.ncbi.nlm.nih.gov/Blast.cgi, accessed on 16 September 2020) against a global assembly (NCBI TSA 330955) containing reads from sexual and apomictic samples reported by Ortiz et al. (2017) [42]. Putative homologous sequences were defined using the following threshold values: score ≥ 100; e-value < 10^−5^ and; ID > 50%. The predicted proteins were checked for containing the conserved AUX/IAA domain (Family PF02309) using the Pfam database (http://pfam.xfam.org/, accessed on 20 September 2020). The molecular weight and isoelectric point (pI) for each sequence were obtained using the Expasy Compute pI/Mw online tool (https://web.expasy.org/compute_pi/, accessed on 20 September 2020). The putative gene structure of the relevant *P. notatum* transcript was reconstructed with the SLPING (www.ncbi.nlm.nih.gov/sutils/splign/splign.cgi, accessed 22 September 2020) online resource [68], using the genomic sequence of *Sorghum bicolor* (Sb08g020580, NC_012877_S_bicolor) as a reference. The gene schemes were drawn with the WormWeb Exon-Intron graphic maker (http://wormweb.org/exonintron, accessed on 9 November 2020). The aux/IAA sequences were aligned using the Clustal Omega tool (https://www.ebi.ac.uk/Tools/msa/clustalo/, accessed on 20 November 2020) and phylogenetic analyses were carried out applying the neighbor-joining method with 1000 bootstrap replications using the MEGA X 10.1.7 software [46]. Associations were considered significant when clades received >50% of bootstrap support.

### 4.3. Expression Analyses of the PnIAA30 in Reproductive and Vegetative Tissues

Total RNA was extracted from spikelets, leaves and roots of both sexual and apomictic plants with the TRIzol (Invitrogen) method [69]. To eliminate possible DNA contamination, RNA samples were treated with RQ1 RNase-Free DNase (Promega Cat. #M6101) following the recommendations of the supplier. Spikelets were collected at three steps of development following Laspina et al.’s developmental calendar (2008) [70], i.e., premeiosis/meiosis, anthesis and two–three days after anthesis. cDNAs were synthesized from 1 μg of total RNA using SuperScript™ II Reverse Transcriptase (Invitrogen/Life Technologies, Cat. No. 18064-022) following the manufacturer’s instructions. PCR primers for the target (*PnIAA30*; NCBI GFMI02021163.1) and the reference (β-*tubulin*; NCBI GFMI02021278 and *GPDH* NCBI GFMI02003604.1) genes were designed with Primer 3 v.0.4.0 (http://bioinfo.ut.ee/primer3-0.4.0/primer3/, accessed on 23 November 2020) [71] (Table S4). *PnIAA30* primers specificity was examined by in silico PCR amplifications using all identified transcripts carrying the AUX/IAA motive as templates with the online tool http://insilico.ehu.eus/PCR, accessed on 25 November 2020. In each cDNA sample, PCR amplification of the target and the reference genes was carried out, including non-transcribed RNA (minus RT) and water (negative) controls to assess cDNA purity (Figure S7). Additionally, PCR amplification of the target and the *GDPH* gene with primers flanking two adjacent exons was routinely performed to control the absence of DNA contamination in cDNA preparations (Figure S7). The reactions were carried out in a 25 μL final volume containing 100 ng of cDNA 1 U of Taq DNA polymerase (INBIO Highway, Tandil, Argentina), 1.5 mM of MgCl_2_, 0.2 µM of dNTPs and 0.2 µM of each primer, in a T100 Thermal Cycler (BIORAD) programed as follows: an initial denaturation step 5 min at 94 °C, followed by 33 cycles of 30 s at 94 °C, 1 min at 59 °C, 40 s at 72 °C. qRT-PCR amplifications were carried out following the general protocol proposed by Pfaffl (2004) [72] in 25-μL reactions containing 200 nM of each of the gene-specific primers, 1 × qPCR Real Mix (Biodynamics, Buenos Aires, Argentina) and 20 ng cDNA. Amplifications were carried out in a Rotor-Gene Q thermocycler (Qiagen) programmed to 2 min at 94 °C followed by 35 cycles of 15 s at 94 °C, 30 s at 57 °C and 40 s at 72 °C. The amplification products of target and reference genes were verified in 2% agarose gels stained with 1 µg/mL ethidium bromide and by constructing melting curves through heating samples to 94 °C for 2 min, cooling down to 70 °C for one min and then increasing the temperature from 70 to 95 °C at the rate of 0.2 °C per 10 s with continuous measurement of the fluorescence (Rotor-Gene Q User Manual, Qiagen). The amplification reactions of *PnIAA30*, *β tubulin* and *GDPH* fragments showed the expected products of 109, 157 and 194 pb, respectively (Figure S7). Melting curve analyses showed a single peak at 85.8, 88.0 and 80.5 °C, respectively (not shown). All qRT-PCR experiments were carried out using at least three biological replicas (i.e., independent RNA extractions of each sample), and each determination was performed utilizing at least three technical replicas, including negative controls (water controls) for the target and the reference genes. The *PnIAA30* expression values were normalized using the *β*-*tubulin* or *GDPH* as reference genes [61]. The quantification of the relative expression was determined using the ΔCt method [73] and/or the REST-RG software, version REST2009_2.0.11 (Corbett Life Sciences, Mortlake, NSW, Australia). 

### 4.4. In Situ Hybridization Experiments

The spikelets were fixed in 4% paraformaldehyde, 0.25% glutaraldehyde, 0.01 M phosphate buffer (pH 7.2), dehydrated in an ethanol/xylol series and embedded in paraffin following the protocol described by Siena et al. (2014) [62]. The samples were then sliced in cross or sagittal sections of 10-μm thick with a Minot microtome and placed onto slides pre-treated with 100 μg mL^−1^ poly-L-lysine. The paraffin was removed with an xylol/ethanol series. A *PnIAA30*-specific fragment of 503 bp (Figure 2a) amplified from cDNA of the Q4117 genotype (using primers IAA30_probeF and IAA30_probeR, Table S4) was used as a probe. The 503 bp *PnIAA30* fragment was cloned into a pGEM^®^-T easy vector, and the recombinant plasmid was used for transforming DH5-alpha *E. coli*-competent cells. The plasmids were purified using the Wizard Plus SV Mini-preps DNA Purification System (Promega, Madison, WI, USA). The cloned fragment was sequenced by Macrogen, Seoul, Korea, to verify both its integrity and orientation. The insert was amplified from the plasmid using M13 forward and reversed primers and used as the template for sense (T7) and antisense (SP6) RNA probe synthesis and labeling using the Roche DIG RNA labeling kit (SP6/T7) (Roche, Basel, Switzerland). Prehybridization was carried out at 37 °C for 10 min in 0.05 M Tris–HCl buffer (pH 7.5) containing 1 μg mL^−1^ proteinase K. Hybridization was performed for 12 h at 37 °C in a buffer containing 10 mM Tris–HCl (pH 7.5), 300 mM NaCl, 50% deionized formamide, 1 mM EDTA (pH 8), 1 × Denhardt’s solution, 10% dextran sulfate, 600 ng mL^−1^ total RNA and 60 ng of the corresponding probe. Detection was performed following the DIG detection kit’s (Roche) instructions using anti-DIG AP and NBT/BCIP as substrates. The sections were analyzed using a Nikon Eclipse E200 microscope equipped with a digital camera. Three different slides carrying 8–9 ovules for each reproductive type were hybridized together in the same hybridization box, and the color reaction was synchronically monitored for all slides. Only hybridization signals showing a consistent pattern were reported as a reliable result. The images of at least 5 ovules from each slide (3 × 5 = 15 total ovules) must have been successfully produced before any conclusion was reached.

### 4.5. Validation of PnIAA30 Antisense RNA Expression

Sense and antisense reads available from the *P. notatum* TSA assembly GenBank GIUR00000000.1 [41] were mapped to the *PnIAA30* transcript using Hisat2 [74], and the read coverage was visualized using the IGV v2.3.90 (Integrative Genomics Viewer, University of California, Oakland, CA, USA) [75] software (http://software.broadinstitute.org/software/igv/, accessed on 10 February 2022) with the option window size 1. Then, we validated the presence of antisense reads by strand-specific cDNA synthesis and PCR amplification according to the protocol reported by Ho et al. (2010) [76]. Briefly, *PnIAA30* antisense strand-specific cDNA was synthesized from RNA samples derived from sexual (Q4188) and apomictic (Q4117) genotypes extracted at anthesis using the ImProm-II Reverse Transcriptase (Promega) with the antisense-specific primer IAA30_probeF (F1) (Table S4) following the recommendations of the supplier. In the same reactions, the sense-specific primers (β-tubulinR and GDPHR) for the *β*-*tubulin* and *GDPH* genes were included (Table S4). The *PnIAA30* antisense strand-specific cDNA was then amplified in PCR reactions containing 50 ng of template, 200 uM of dNTPs, 1.5 mM Cl_2_Mg, 1x Taq buffer, 1.5 U Taq polymerase INBIO (Buenos Aires, Argentina) and 0.2 uM of the specific primer for the target and reference genes (Table S4). The thermocycler conditions for amplifying *PnIAA30* fragments were as follows: 2 min at 95 °C followed by 35 cycles of 30 s at 95 °C, 50 s at 57–58 °C and 1 m at 72 °C, and a final step of 5 m at 72 °C. The PCR products were electrophoresed in a 2% agarose gels in 1x TAE buffer as described above. 

### 4.6. Identification of Putative PnIAA30 Interactors and In Silico Expression Analysis

The STRING database (https://string-db.org/, accessed on 10 June 2021) [49] was used to infer protein–protein interactors for both *OsIAA30* and its *Arabidopsis* ortholog, *AtIAA16*. Then, *P. notatum* transcripts encoding for interactor’s homologs were identified by TBLASTN searches on the annotated stage-specific quantitative flower transcriptome of the species (NCBI BioProject: 511813; accession PRJNA511813) [41]. The expression patterns of the *P. notatum* transcripts were recovered from the differential analysis reported by Podio et al. (2021) [41]. The heat maps of transcript expression were built with the HEATMAPPER (http://www.heatmapper.ca/, accessed on 7 December 2020) online tool [77].

## Supplementary Materials

The following supplemental information are available online at https://www.mdpi.com/article/10.3390/plants11111472/s1, Figure S1: Protein alignment and consensus for the *Paspalum* floral Aux/IAA-predicted protein sequences. Figure S2: Alignment of *PnIAA30* nucleotide and protein sequences from apomictic and sexual genotypes. Figure S3: Expression analysis of *PnIAA30* transcripts in leaves and roots. Figure S4: in situ hybridization of *PnIAA30* sense probe on reproductive tissues of sexual and apomictic individuals. Figure S5: in situ hybridization of *PnIAA30* antisense probe on reproductive tissues of sexual and apomictic individuals. Figure S6: in situ hybridization of negative controls. Figure S7: *PnIAA30*, *β-tubulin* and *GDPH* amplicons used in qRT-PCR assays. Table S1: TBLASTN analysis using the rice IAA/AUX proteins as a query on the reference *P. notatum* floral transcriptome [42]. Table S2: qRT-PCR analysis statistics, Table S3: Analysis of IAA30 (*Oryza sativa*) and IAA16 (*Arabidopsis thaliana*) predicted interactors. Table S4: Primers used in PCR analyses. References [78,79] are cited in the supplementary materials.
