# Supplementary material for: The Auxin-Response Repressor IAA30 Is Down-Regulated in Reproductive Tissues of Apomictic Paspalum notatum
Source: Plants (Basel). 2022 May 31;11(11):1472. doi: 10.3390/plants11111472 (PMC9182604; doi:10.3390/plants11111472)
Supplement: Supplementary file 1 [file plants-11-01472-s001.zip › plants-1422384-supplementary.pdf]

Article

The auxin-response repressor *IAA30* is down-regulated in reproductive tissues of apomictic *Paspalum notatum*. Lorena Adelina Siena, Celeste Antonela Azzaro, Maricel Podio, Juliana Stein, Olivier Leblanc, Silvina Claudia Pessino and Juan Pablo Amelio Ortiz.

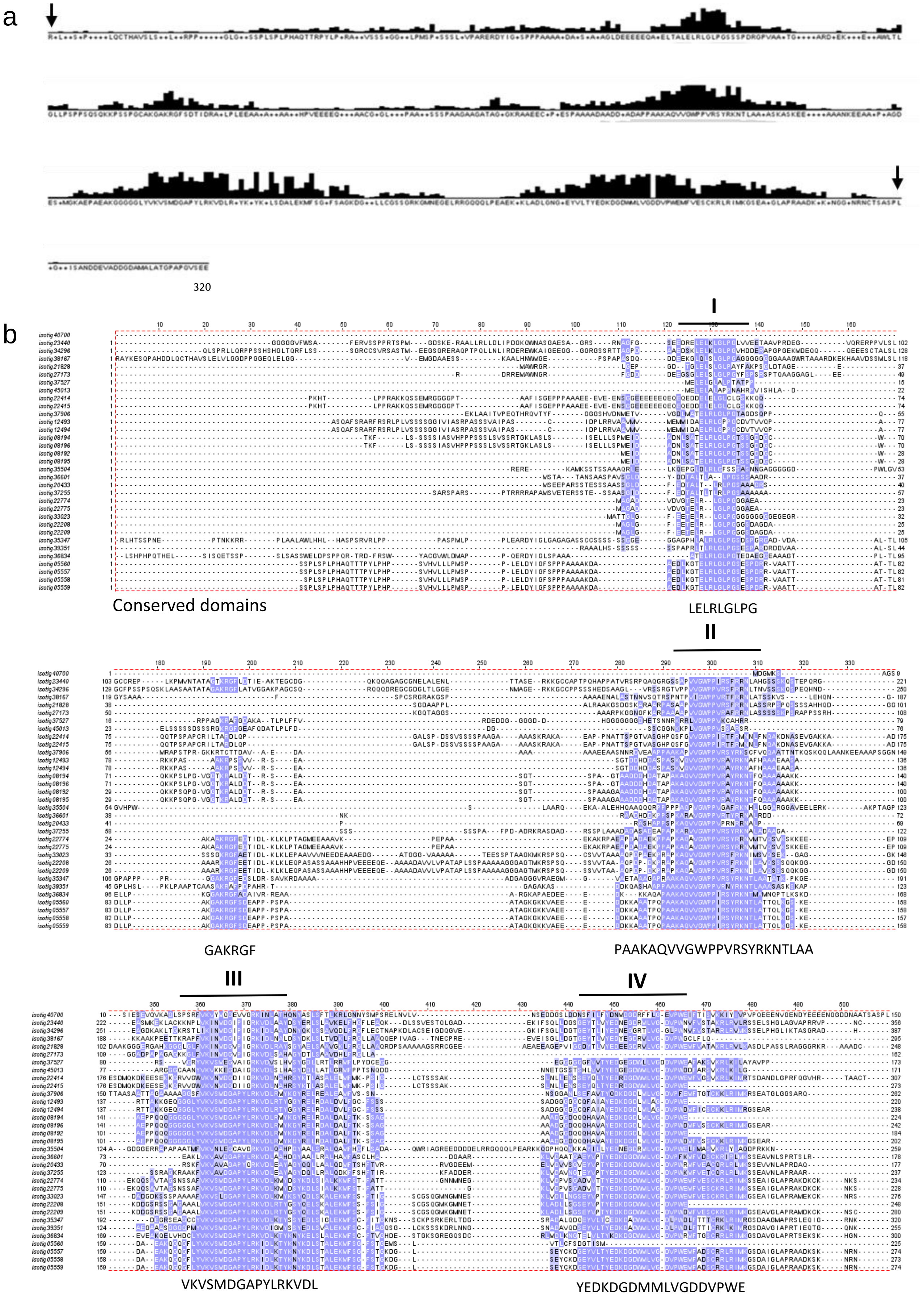

Figure S1. Alignment of predicted *P. notatum* Aux/IAA proteins. **a.** Overview of the alignment obtained with the ClustalO (https://www.ebi.ac.uk/Tools/msa/clustalo/, accessed on 20 November 2020) online tool. The height of the bars indicates the number of identical residues per position. The arrows indicate the region containing conserved domains and shown below. **b.** Alignment of the 33 unique *P. notatum* transcripts coding for AUX/IAA proteins. Domains I to IV are indicated with by roman numerals and solid lines. Conserved residues (present in more than 50% of aligned sequences) are highlighted in color. Gaps (marked with dashes) have been introduced to maximize the alignments. The respective amino acid position is given on the left and right of each sequence.

a.

```
                                                                                 start
PnIAA30_apo    GCCCCCCACCCCCTCCTCTCCCTATCTATTTCTCTCCCATTATTTCCATTGCATTCGCAT    60
PnIAA30_sex    -----------------------ATCTATTTCTCTCCCATTATTTCCATTGCATTCGCAT    37
                                      *************************************

PnIAA30_apo    GCCTTGGCGTTGCAAGCCAAGAACACCAACCAGCAACGAAGCAGCCAAGCAGGGAGGCTA    120
PnIAA30_sex    GCCTTGGCGTTGCAAGCCAAGAACACCAACCAGCGACGAAGCAGCCAAGCAGGGAGGCTA    97
               ********************************** *************************
                                                                 start
PnIAA30_apo    GCAAGCAGAAGGGGCGGTCGAGGATCGGAGGTGGATTGCTTGAGCAATGGCGACGACGGG    180
PnIAA30_sex    GCAAGCAGAAGGGGCGGTCGAGGATCGGAGGTGGATTGCTTGAGCAATGGCGACGACGGG    157
               ************************************************************

PnIAA30_apo    CCTGGGGTTCGAGGAGACGGAGCTCCGGCTGGGCCTGCCCGGCGGCGGCGGCGGAGGCGG    240
PnIAA30_sex    CCTGGGGTTCGAGGAGACGGAGCTCCGGCTGGGCCTGCCCGGCGGCGGCGGC---GGCGG    214
               ****************************************************   *****

PnIAA30_apo    GGGAGGAGAGGGCGAGGGCAGGAGCTCCTCCGGCAAGCGGGGCTTCGCCGAGACCATCGA    300
PnIAA30_sex    GGGAGGAGAGGGCGAGGGCAGGAGCTCCTCCGGCAAGCGGGGCTTCGCCGAGACCATCGA    274
               ************************************************************

PnIAA30_apo    CCTCAAGCTGAAGCTGGAGCCGGCGGCCGTGGTGGTGAACGAGGAGGATGAGGCGGCGGC    360
PnIAA30_sex    CCTCAAACTGAAGCTGGAGCTGGCGGCCGTGGTGGTGAACGAGGAGGATGAGGCGGCGGC    334
               ****** ************* ***************************************

PnIAA30_apo    GGAGGACGGTGCCACGGGTGGTGGCGTGGCCGCCGCCGCGGCGACGGAGGAATCGTCCCC    420
PnIAA30_sex    GGAGGACGGTGCCACGGGTGGTGGCGTGGCCGCCGCCGCGGCGACGGAGGAATCGTCCCC    394
               ************************************************************

PnIAA30_apo    CACCGCGGCCGGGAAGATGAAGAGGTCCCCGAGCCAGTGCAGCGTCGTCACCGCCGCCGC    480
PnIAA30_sex    CACCGCGGCCGGGAAGATGAAGAGGTCCCCGAGCCAGTGCAGCGTCGTCACCGCCGCCGC    454
               ************************************************************

PnIAA30_apo    CGCCCAGCCCGACCCCGCCGAGAAGCCGCGCGCCCCCAAGGCTCAGGTGGTGGGATGGCC    540
PnIAA30_sex    CGCCCAGCCCGACCCCGCCGAGAAGCCGCGCGCCCCCAAGGCTCAGGTGGTGGGATGGCC    514
               ************************************************************

PnIAA30_apo    GCCGGTGCGGTCCTTCCGGAAGAACATCATGTCCGTGCAGTCCGAGAAAGGCGCCGGCGG    600
PnIAA30_sex    GCCGGTGCGGTCCTTCCGGAAGAACATCATGTCCGTGCAGTCCGAGAAAGGCGCCGGCGG    574
               ************************************************************

PnIAA30_apo    CAAGGACGCCGACGGCGACAAGTCCAGCTCGCCGGCGGCCGCCGCGTTGGTCAAGGTGAG    660
PnIAA30_sex    CAAGGACGCCGACGGCGACAAGTCCAGCTCGCCGGCGGCCGCCGCGTTTGTCAAGGTGAG    634
               ************************************************ ***********

PnIAA30_apo    CTTGGACGGCGCGCCGTACCTGCGCAAGGTGGACCTCAAGATGTACAAGAGCTACCAGGA    720
PnIAA30_sex    CTTGGACGGCGCGCCGTACCTGCGCAAGGTGGACCTCAAGATGTACAAGAGCTACCAGGA    694
               ************************************************************

PnIAA30_apo    CCTGTCCAAGGCGCTCGAGAAGATGTTCAGCTCCTTCACCATCGGAAGCTGTGGGTCTCA    780
PnIAA30_sex    CCTGTCCAAGGCGCTCGAGAAGATGTTCAGCTCCTTCACCATCGGAAGCTGTGGGTCTCA    754
               ************************************************************

PnIAA30_apo    GGGGATGAACGGCATGAACGAGAGCAAGCTGGTGGATCTGCTCAACGGCTCTGAGTACGT    840
PnIAA30_sex    GGGGATGAACGGCATGAACGAGAGCAAGCTGGTGGATCTGCTCAACGGCTCTGAGTACGT    814
               ************************************************************

PnIAA30_apo    GCCGACCTACGAGGACAAGGACGGCGACTGGATGCTCGTCGGCGACGTGCCGTGGGAGAT    900
PnIAA30_sex    GCCGACCTACGAGGACAAGGACGGCGACTGGATGCTCGTCGGCGACGTGCCGTGGGAGAT    874
               ************************************************************
PnIAA30_apo    GTTCGTCGAATCGTGCAAGCGCCTTCGGATCATGAAAGGATCAGAAGCCATTGGCCTCGC    960
PnIAA30_sex    GTTCGTCGAATCGTGCAAGCGCCTTCGGATCATGAAAGGATCAGAAGCCATTGGCCTCGC    934
               ************************************************************
                                                  stop
PnIAA30_apo    ACCAAGGGCCATGGAGAAATGCAAGAACAGAAGCTGAGGAGAAGATGGGCGACGCATGCA    1020
PnIAA30_sex    ACCAAGGGCCATGGAGAAATGCAAGAACAGAAGCTGAGGAGAAGATGGGCGACGCATGCA    994
               ************************************************************
```

```
PnIAA30_apo          TCTGAATTATTCTGAGTACCATGGATCTCATCGGATGCAAGAAGGACCACAGCCTCCACA    1080
PnIAA30_sex          TCTGAATTATTCTGAGTACCATGGATCTCATCGGATGCAAGAAGGACCACAGCCTCCACA    1054
                     ************************************************************

PnIAA30_apo          GCTCTGCTCGTCTCTGTATCATACTCGTCCCTGTGTTCTCCAAGCCTCTACTACCACCTC    1140
PnIAA30_sex          GCTCTGCTCGTCTCTGTATCATACTCGTCCCTGTGTTCTCCAAGCCTCTACTACCACCTC    1114
                     ************************************************************

PnIAA30_apo          CTGATAATATCAGTGGAGTCATTTTGGTGTGTCAACTGTCCTATCTGTTCCAGTGAGTCA    1200
PnIAA30_sex          CTGATAATATCAGTGGAGTCATTTTGGTGTGTCAACTGTCCTATCTGTTCCAGTGAGTCA    1174
                     ************************************************************

PnIAA30_apo          GTAAGACAGTCAGTCAGTGTTCGAGTCTCATCTTGTCATATATACTTCTACTACGAAAGA    1260
PnIAA30_sex          GTAAGACAGTCAGTCAGTGTTCGAGTCTCATCTTGTCATATATACTTCTACTACGAAAGA    1234
                     ************************************************************

PnIAA30_apo          GAGAAGCCATGAACGTGTACCTGTCCTATAACAGTGTCTTTTTGCTCTACCTGTTCCCTT    1320
PnIAA30_sex          GAGAAGCCATGAACGTGTACCTGTCCTATAACAGTGTCTTTTTGCTCTACCTGTTCCCTT    1294
                     ************************************************************

PnIAA30_apo          TTTTTCCACAAAATGCAAAGGCACCCTGCTCCTAATCAATCAAGGGGGCCCTTTTGCATC    1380
PnIAA30_sex          TTTTTCCACAAAATGCAAAGGCACCCTGCTCCTAATCAATCAAGGGGGCTCTTTTGCATC    1354
                     *************************************************▲**********

PnIAA30_apo          TTGTCGATCAAAAGTTGTTTAATTAATATTACCATGGTGTGCCACTGGGACAAAATAATT    1440
PnIAA30_sex          TTGTCGATCAAAAGTTGTTTAATTAATATTACCATGGTGTGCCACTGGGACAAAATAATT    1414
                     ************************************************************

PnIAA30_apo          GTTGCAGGCTGTTAAAAAGCTGTATTGTATTATCATCTCCTCTCCCTTCCTTCTTTGTTC    1500
PnIAA30_sex          GTTGCAGGCTGTTAAAAAGCTGTATTGTATTATCATCTCCTCTCCCTTCCTTCTTTGTTC    1474
                     ************************************************************

PnIAA30_apo          TTTCACCATATTGGTGTTAACTGTTCCATGCAGTAGCTAGGATTACAAACTCCATGATGA    1560
PnIAA30_sex          TTTCACCATATTGGTGTTAACTGTTCCATGCAGTAGCTAGGATTACAAACTCCATGATGA    1534
                     ************************************************************

PnIAA30_apo          AAGATATTGAGATGCCTTGTTTG------                                   1583
PnIAA30_sex          AAGATATTGAGATGCCTTGTTTTGTGGTG                                   1563
                     **********************
```

b.

```
PnIAA30_apo          MATTGLGFEETELRLGLPGGGGGGGGGEGEGRSSSGKRGFAETIDLKLKLEPAAVVVNEE    60
PnIAA30_sex          MATTGLGFEETELRLGLPGGGG-GGGGEGEGRSSSGKRGFAETIDLKLKLELAAVVVNEE    59
                     ********************** **************************** ********

PnIAA30_apo          DEAAAEDGATGGGVAAAAATEESSPTAAGKMKRSPSQCSVVTAAAAQPDPAEKPRAPKAQ    120
PnIAA30_sex          DEAAAEDGATGGGVAAAAATEESSPTAAGKMKRSPSQCSVVTAAAAQPDPAEKPRAPKAQ    119
                     ************************************************************

PnIAA30_apo          VVGWPPVRSFRKNIMSVQSEKGAGGKDADGDKSSSPAAAAFVKVSLDGAPYLRKVDLKMY    180
PnIAA30_sex          VVGWPPVRSFRKNIMSVQSEKGAGGKDADGDKSSSPAAAAFVKVSLDGAPYLRKVDLKMY    179
                     ************************************************************

PnIAA30_apo          KSYQDLSKALEKMFSSFTIGSCGSQGMNGMNESKLVDLLNGSEYVPTYEDKDGDWMLVGD    240
PnIAA30_sex          KSYQDLSKALEKMFSSFTIGSCGSQGMNGMNESKLVDLLNGSEYVPTYEDKDGDWMLVGD    239
                     ************************************************************

PnIAA30_apo          VPWEMFVESCKRLRIMKGSEAIGLAPRAMEKCKNRS                            276
PnIAA30_sex          VPWEMFVESCKRLRIMKGSEAIGLAPRAMEKCKNRS                            275
                     ************************************
```

Figure S2. Alignment of nucleotide and amino acid sequences of *PnIAA30* expressed in the sexual and apomictic floral transcriptomes of *P. notatum.* **a)** alignment of *PnIAA30* nucleotide sequences expressed in the apomictic (Q4117) and sexual (Q4188) floral transcriptomes. Arrow heads show one indel at the 3′ region and SNPs scattered along sequences. **b)** predicted proteins from apomictic and sexual transcripts.

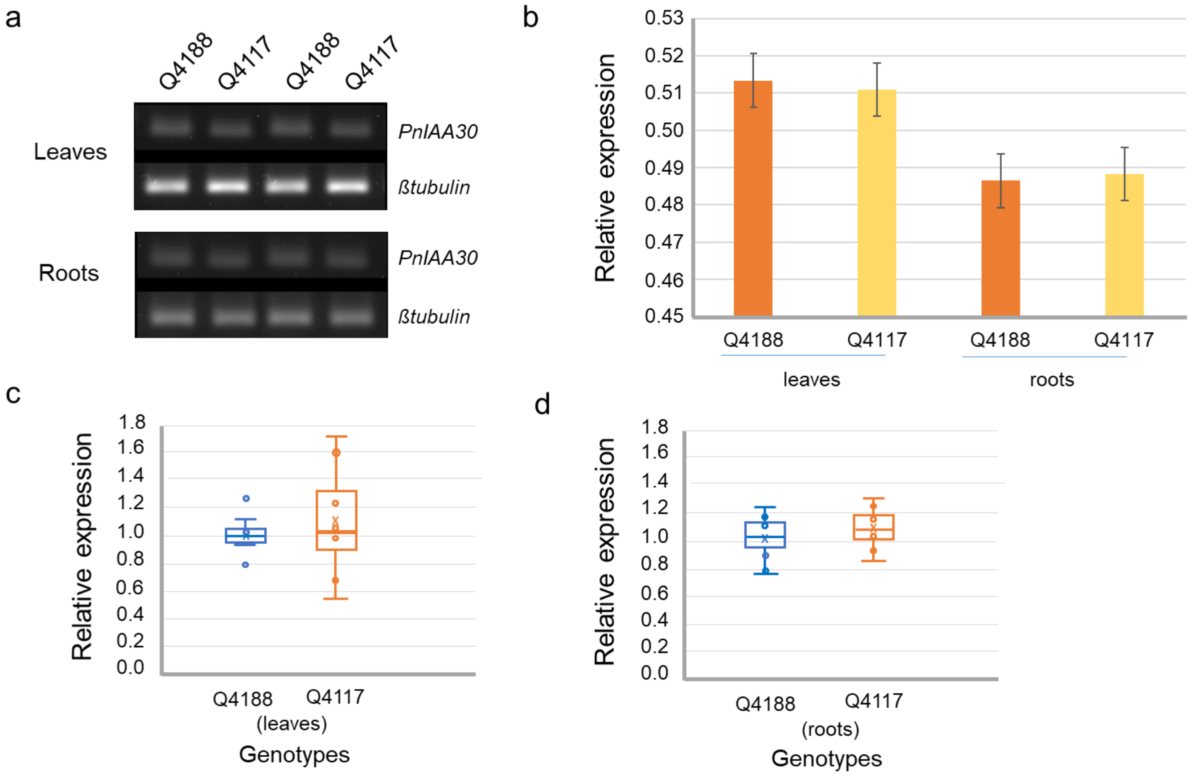

Figure S3. Relative expression analyses of *PnIAA30* in leaves and roots of sexual (Q4188) and apomictic (Q4117) genotypes of *P. notatum.* **a)** agarose gel stained with SYBER-safe showing the amplification products of *PnIAA30 and ß tubulin* expressed in leaves and roots run by duplicate. **b)** semi-quantitative analysis of amplification bands carried out with the Image J software [78]. Vertical bars indicate standard deviations. **c** and **d**) Box plots of qRT-PCR analysis of samples from leaves (**c**) and roots (**d**). In qRT-PCR graphics each box shows the variance of relative expression values derived from three independent experiments run in triplicate (n =9) from three biological replicas of each sample. In each box lower boundary indicating the 25th percentile, the line within the box indicating the median, the x representing the mean, and the upper edge marking the 75th percentile. Upper and lower whisker caps indicate the 95th and 5th percentiles. Outliers are indicated with circles.). RT-PCR reactions were carried out in 25 µl using 100 ng of cDNA (derived from young leaves, roots and spikelets collected two days after anthesis), 1 U of Taq DNA polymerase (INBIO Highway, Tandil, Argentina), 1.5 mM of MgCl2, 0.2 µM of dNTPs and 0.2 µM of each primer, using a T100 Thermal Cycler (BIORAD). Amplification reactions consisted of an initial denaturation step 5 min at 94 °C, followed by 33 cycles of 30 s at 94 °C, 1 min at 59 °C, 40 s at 72° C. Amplification fragments of the β-TUB or G6PDH transcripts were used as normalizers. The amplifications products were separated in 2% agarose gels stained with 1:10,000 SYBR® Safe fluorophore (Promega Cat. # S-33102), in TAE 1X (v/v) buffer using a Mupid®-One cell (Ref. MU-0041-) electrophoresis tank for 1 h 20 min at 50 V. Images were taken with Molecular Imager® Gel Doc ™ XR System using the SYBR ® Safe option. The semi-quantification of expression of amplification products bands was carried out using the ImageJ software (https://imagej.nih.gov/ij/download.html) (Abramoff et al., 2004) [78]. The expression value of the target gene was normalized with the expression values of the reference genes [79].

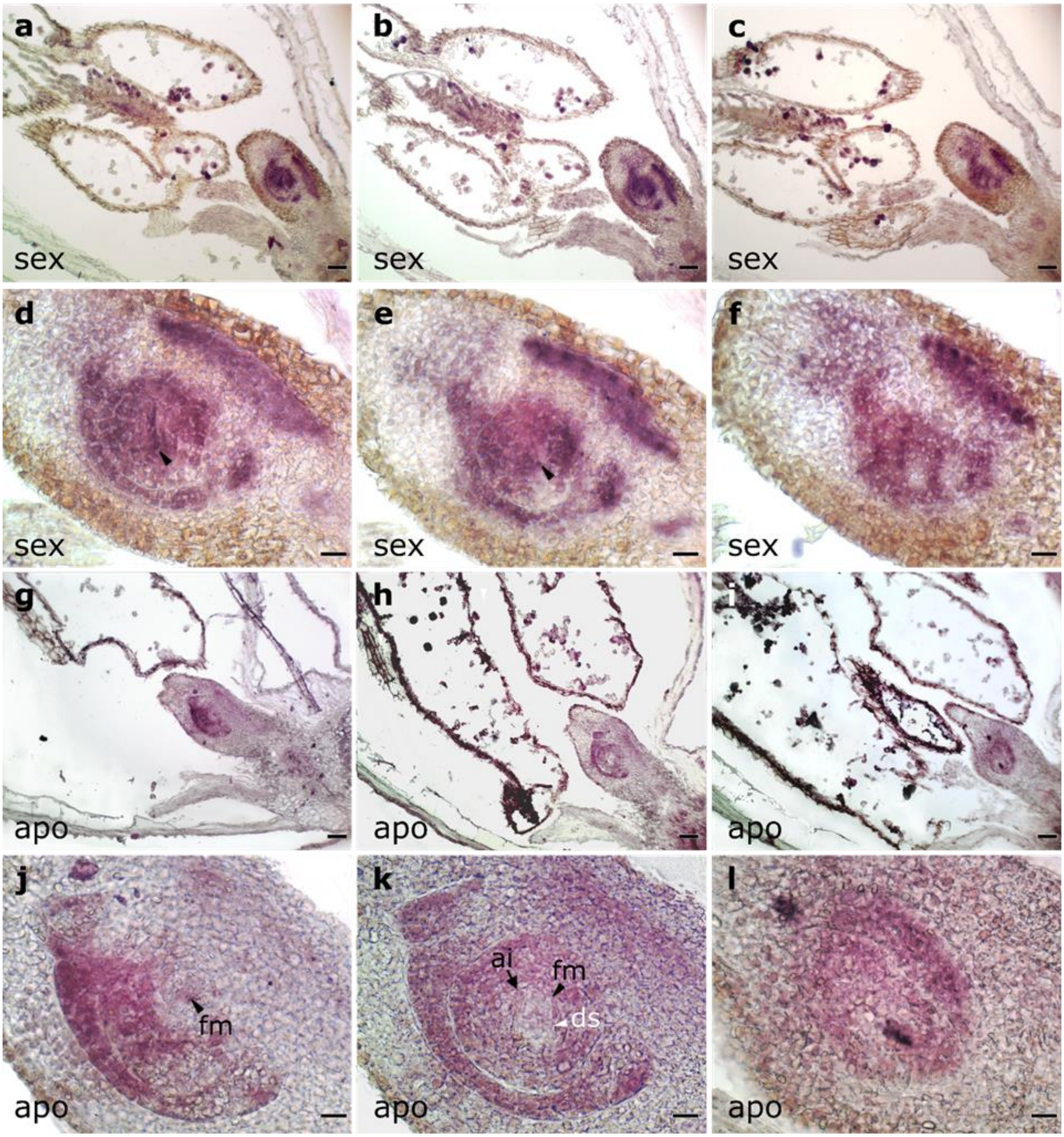

Figure S4. RNA *in situ* hybridization of *PnIAA30* transcripts with the antisense probe in reproductive tissues of sexual (Q4188) and apomictic (Q4117) *P. notatum* individuals at the stage of premeiosis/meiosis. **a-f** and **g-i**) images of adjacent sections a sexual (**a-f**) and apomictic (**g-l**) floral tissues hybridized with the sense probe showing the expression localization of *PnIAA30* sense transcript. **a-c** and **g-i**) images taken with 10x objective lens showing the contrasting hybridization signals between reproductive and non-reproductive tissues. Note the strong staining within primordium and anthers compared with the surrounding somatic tissues. **d-f** and **j-l**) images of ovules taken with 40x objective lens showing the localization of *PnIAA30* transcript within the primordium of sexual **(d-f)** and apomictic (**j-l**) ovules. Pictures shown in **j** and **k** are the same as included in Figure 4. **ai**: apospory initials, **apo**: ovules of Q4117, **ds**: degenerating spores, **fm:** functional megaspore cell, **sex:** ovules of Q4188. Bars: **d-f** and **j-l** = 10 μm; **a-c** and **j-l** = 25 μm.

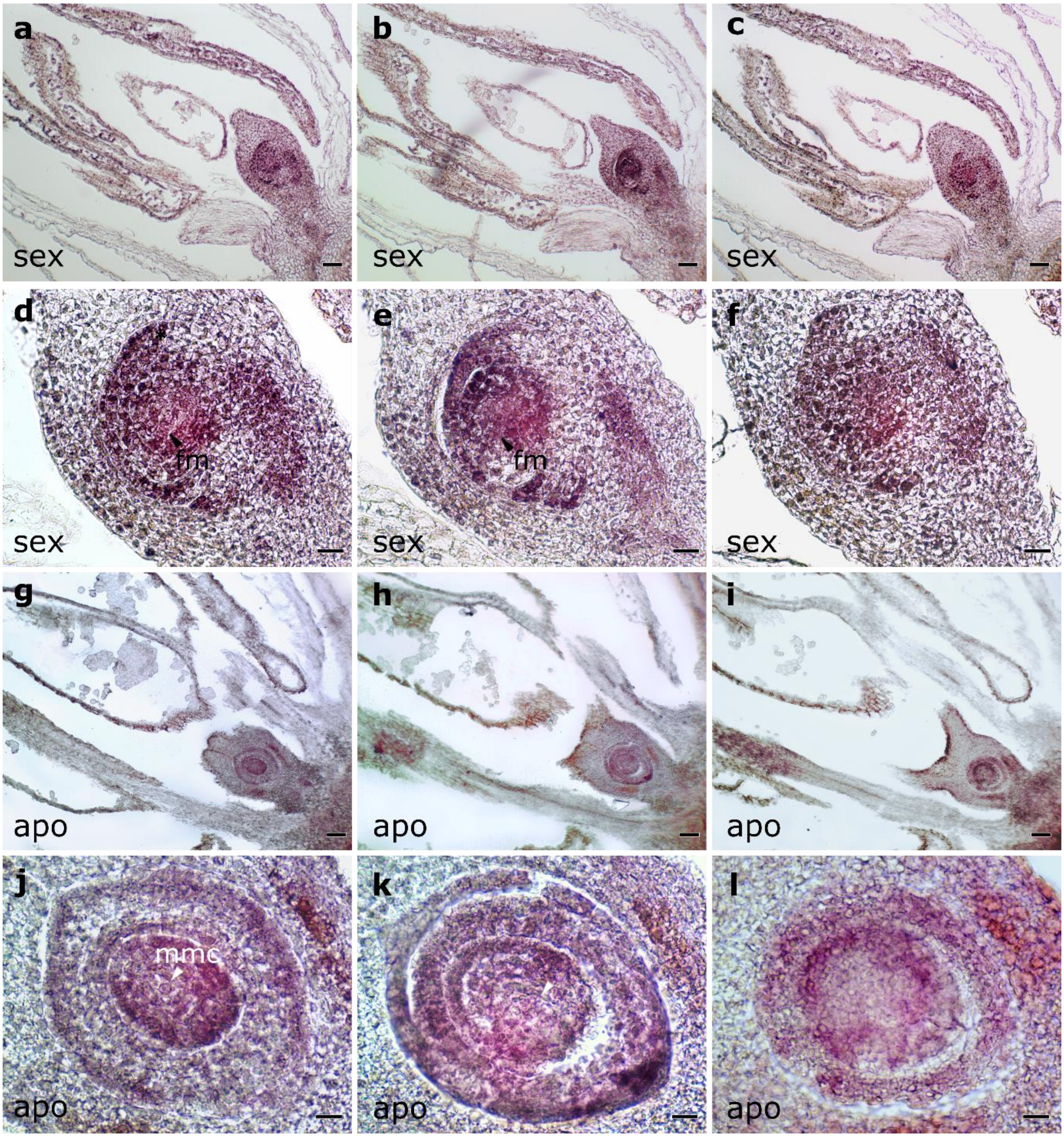

Figure S5. RNA *in situ* hybridization of *PnIAA30* transcripts with the sense probe in reproductive tissues of sexual (Q4188) and apomictic (Q4117) *P. notatum* individuals at the stage of premeiosis/meiosis. **a-f** and **g-i**) images of adjacent sections a sexual (**a-f**) and apomictic (**g-l**) floral tissues hybridized with the sense probe showing the expression localization of *PnIAA30* antisense transcript. **a-c** and **g-i**) images taken with 10x objective lens showing the contrasting hybridization signals between reproductive and non-reproductive tissues. **d-f** and **j-l**) images of ovules taken with 40x objective lens showing the localization of *PnIAA30* antisense transcript within the primordium of sexual **(d-f)** and apomictic (**j-l**) ovules. Pictures shown in **j** is the same as included in Figure 4. **apo**: ovules of Q4117, **mmc:** megaspore mother cell, **sex:** ovules of Q4188. Bars: **d-f** and **j-l** = 10 μm; **a-c** and **j-l** = 25 μm.

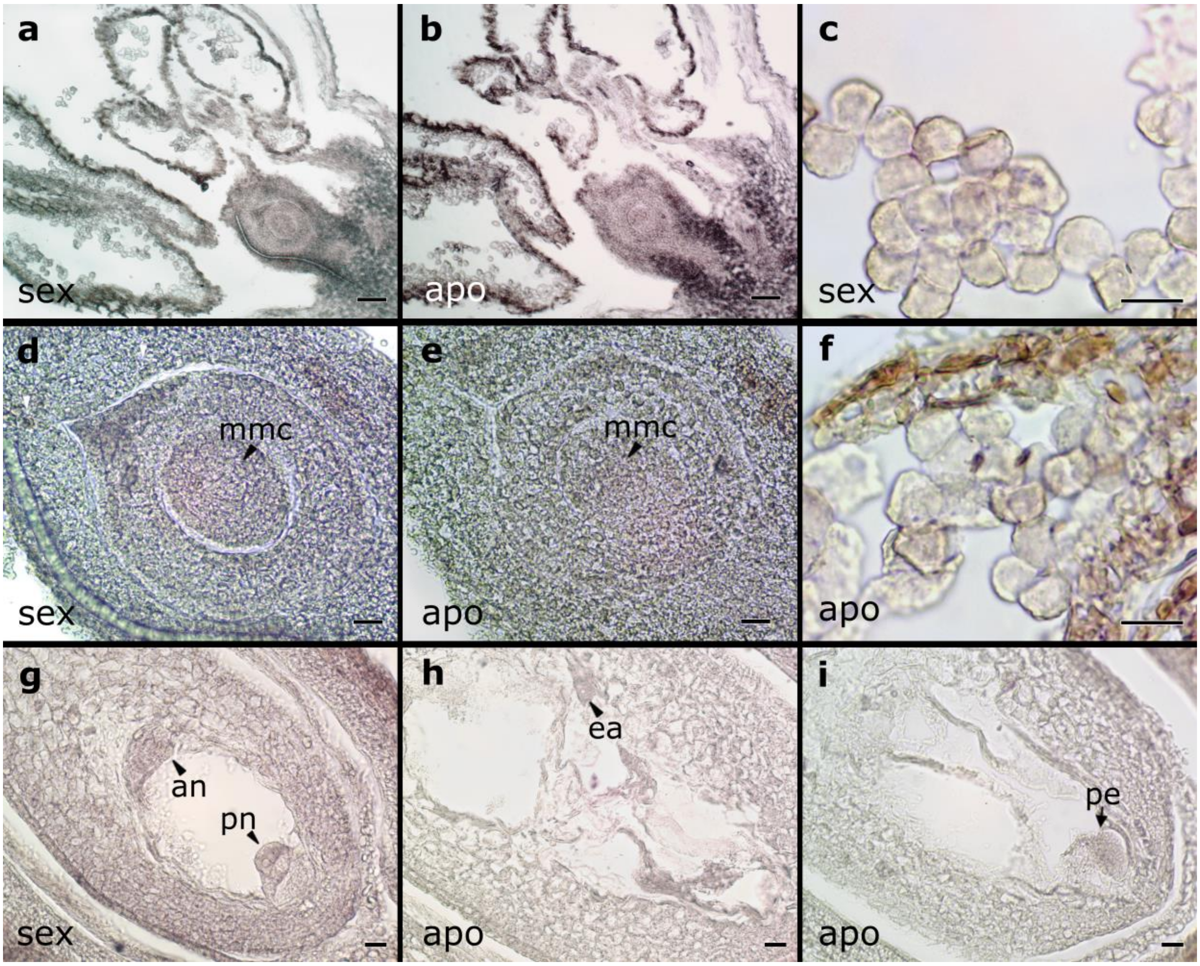

Figure S6. RNA *in situ* hybridization on reproductive tissues of sexual (Q4188) and apomictic (Q4117) *P. notatum* individuals negative (water) controls. **a-b)** images of sexual and apomictic samples taken with 10x lens showing lack of staining**.** **c** and **f**: microspores of sexual (**c**) and apomictic (**f**) flowers. **d-e**: ovules of sexual and apomictic genotypes at the stage of MMC. **g-i:** sexual (**g**) and aposporous (**h-i**) embryo sacs at the stage of anthesis. **apo:** ovules of Q4117, mmc: megaspore mother cell, **sex:** ovules of Q4188. Bars: d-f and j-l = 10 μm; a-c and j-l = 25 μm.

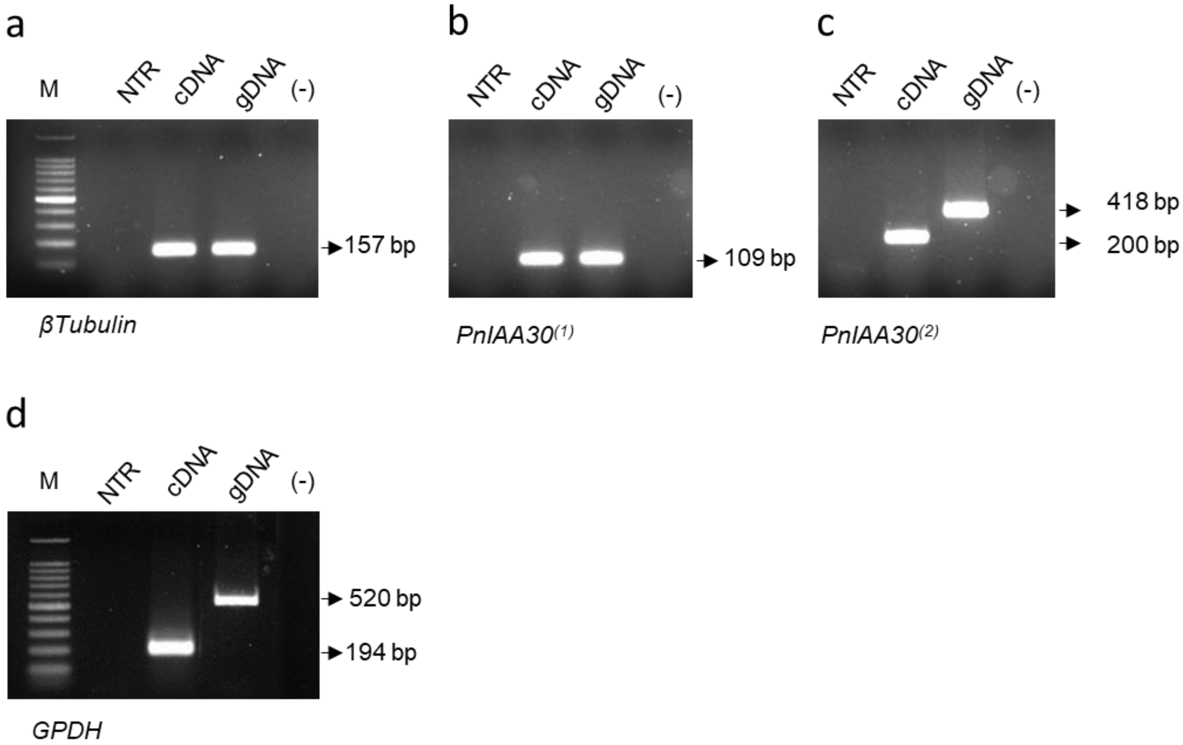

Figure S7. PCR amplification of fragments used in qRT-PCR experiments. **a)** *β-tubulin* amplicon (157 bp) generated with primers *β-tubulin*F and *β-tubulin*R; **b)** *PnIAA30 3′* fragment (109 bp) amplified with primers RTPnIAA30F and RTPnIAA30R, **c)** *PnIAA30 3′* fragment generated with primers RTPnIAA30IF and RTPnIAA30IR which pair at the borders of the 4th and 5th exons, respectively, and **d)** *GDPH* fragment amplified with primers GPDHF and FDPHR which pair at the borders of the 9th and 11th exons. **M:** molecular weight marker, **NTR:** non-transcribed RNA; **cDNA:** complementary DNA, **gDNA:** genomic DNA, **(-)**: negative (non-template water control).
