# Supplementary material for: The Auxin-Response Repressor IAA30 Is Down-Regulated in Reproductive Tissues of Apomictic Paspalum notatum
Source: Plants (Basel). 2022 May 31;11(11):1472. doi: 10.3390/plants11111472 (PMC9182604; doi:10.3390/plants11111472)
Supplement: Supplementary file 1 [file plants-11-01472-s001.zip › Table S4.pdf]

Table S4. Primers used for qRT-PCR and RT-PCR amplification reactions:

Primer name	5′-3′ sequence	Fragment	Length of amplification product/s (bp)
RTPnIAA30F<br>RTPnIAA30R	TGGTGTGTCAACTGTCCTATCTG<br>TTCATGGCTTCTCTCTTTCGTAG	*PnIAA30* fragment used in qRT-PCR assays	109[1]
RTPnIAA30IF<br>RTPnIAA30IR	ATGAAAGGATCAGAAGCCATTG<br>GTAGAGGCTTGGAGAACACAGG	*PnIAA30* fragment spanning between adjacent exons	200[1], 418[2]
IAA30_probeF<br>IAA30_probeR	CGACGCATGCATCTGAATTA<br>TGCATGGAACAGTTAACACCA	*PnIAA30* fragment used as a probe in in situ hybridization experiments	503[1,2]
β-tubulinF<br>*β*-tubulinR	GTGGAGTGGATCCCCAACAA<br>AAAGCCTTCCTCCTGAACATGG	*β-tubulin* fragment used in qRT-PCR assays as normalizer	157[1,2]
GPDHF<br>GPDHR	CATCAGAGATGAGAAAGTCAAGGTT<br>AGCTTTAAGAATGAAAGGAACACCT	*GDPH* fragment spanning between adjacent exons used in qRT-PCR assays as normalizer	194[1], 520[2]

[1]using cDNA as template, [2]usign genomic DNA as template
